# Identification of Cuproptosis-Associated Prognostic Gene Expression Signatures from 20 Tumor Types

**DOI:** 10.3390/biology13100793

**Published:** 2024-10-03

**Authors:** Ednah Ooko, Nadeen T. Ali, Thomas Efferth

**Affiliations:** 1 Laboratory of Cell Biology, Center for Cancer Research, National Cancer Institute, National Institutes of Health, Bethesda, MD 20892, USA; ednah.ooko@nih.gov; 2 Department of Biological Sciences, School of Natural and Applied Sciences, Masinde Muliro University of Science and Technology, Kakamega 190-50100, Kenya; 3 Department of Pharmaceutical Biology, Institute of Pharmaceutical and Biomedical Sciences, Johannes Gutenberg University, Staudinger Weg 5, 55128 Mainz, Germany; neltayeb@uni-mainz.de

**Keywords:** cancer, gene signatures, prognostic factors, RNA-sequencing, signaling pathways, survival time

## Abstract

**Simple Summary:**

Non-apoptotic modes of cell death have gained increasing attention in the past few years. Cuproptosis is a copper-dependent cell death mechanism that is involved in numerous diseases, including cancer. The relevance of cuproptosis for the survival times of cancer patients is still incompletely understood. We have investigated cuproptosis-related genes based on their mRNA expression and statistical relationship to the survival times of patients by using Kaplan–Meier statistics. Further, we have investigated the possible interactions of the genes with signaling networks. Our study has shown that cuproptosis may play an important role in hepatocellular carcinoma, renal clear cell carcinoma, papillary renal cell carcinoma, and lung adenocarcinoma. We identified gene signatures consisting of nine to twenty-one genes in the above four tumor types, and we have shown that a high mRNA expression of 63/124 cuproptosis-associated genes significantly correlated with shorter survival times of cancer patients.

**Abstract:**

We investigated the mRNA expression of 124 cuproptosis-associated genes in 7489 biopsies from 20 different tumor types of The Cancer Genome Atlas (TCGA). The KM plotter algorithm has been used to calculate Kaplan–Meier statistics and false discovery rate (FDR) corrections. Interaction networks have been generated using Ingenuity Pathway Analysis (IPA). High mRNA expression of 63 out of 124 genes significantly correlated with shorter survival times of cancer patients across all 20 tumor types. IPA analyses revealed that their gene products were interconnected in canonical pathways (e.g., cancer, cell death, cell cycle, cell signaling). Four tumor entities showed a higher accumulation of genes than the other cancer types, i.e., renal clear cell carcinoma (*n* = 21), renal papillary carcinoma (*n* = 13), kidney hepatocellular carcinoma (*n* = 13), and lung adenocarcinoma (*n* = 9). These gene clusters may serve as prognostic signatures for patient survival. These signatures were also of prognostic value for tumors with high mutational rates and neoantigen loads. Cuproptosis is of prognostic significance for the survival of cancer patients. The identification of specific gene signatures deserves further exploration for their clinical utility in routine diagnostics.

## 1. Introduction

Metals play essential roles in human physiology, e.g., the transition metals iron (Fe), copper (Co), manganese (Mn), and molybdenum (Mo) act as cofactors of metalloenzymes. Redox reactions catalyzed by metal ions are involved in electron transfer processes that are tightly controlled to maintain metal homeostasis in the body; this is the case with Mo [1]. In general, metals are involved in the activation of normal physiological functions, e.g., immune reactions, electron transfer during mitochondrial respiration, xenobiotic metabolism, and oxygen transport in blood and tissues as carried out by Fe, Co, and Mn [2,3]. Fe is involved in the regulation of enzymatic activity, oxygen transmission, and DNA repair; Co is crucial for cytochrome c oxidase in the mitochondria; Mn is important for control of heart function and blood pressure regulation; Mo is an important cofactor for several enzyme activities. Upon disturbances of this balance, reactive oxygen species (ROS) are generated (mainly by the Fenton reaction) that are harmful to nucleophilic molecules such as DNA, proteins, and biomembranes [4]. Hence, transition metal ion deficiency can cause diseases, e.g., iron deficiency in anemia or chronic kidney disease; copper deficiency in anemia, brain disease and heart disease; manganese deficiency in epilepsy and diabetes; molybdenum deficiency in encephalopathy and intractable seizures [5,6].

On the other hand, an excess of metal ions can be toxic. Disrupted metal homeostasis and enhanced ROS generation lead to oxidative stress that may override the body’s antioxidant protective capacity causing acute or chronic toxicity [4]. However, redox inactive metals (e.g., cadmium (Cd), lead (Pb), or arsenic (As)) can also exert toxicity by binding thiol-groups and depleting glutathione [7]. This results in a reduction of the cellular energy that the body would get because of the interruption of the flow of electrons caused by the redox inactive metal. A main source for contamination with metals (lead, cadmium, nickel, mercury, arsenic, etc.) is drinking water [8]. An exception is the redox inert transition metal zinc which counteracts diseases by reducing oxidative stress [9,10,11].

Occupational and environmental exposure to metals can be carcinogenic, not only by causing oxidative stress and DNA damage but also by epigenetic mechanisms and alterations in signal transduction pathways [12,13]. Once a tumor has developed, metals such as iron also contribute to typical hallmarks of tumor progression, e.g., metastasis and angiogenesis [14]. Recent interest has focused on another transition metal, copper. Like iron, copper generates ROS and contributes to cancer growth, epithelial to mesenchymal transition, metastasis, and angiogenesis [15,16,17].

Despite the role of iron and copper in cell proliferation, they also sense cell death. Copper-dependent growth has been termed cuproplasia, while the mechanisms of these two transition metals have been termed ferroptosis and cuproptosis, respectively [18,19,20,21]. Both forms of the programed cell are involved in numerous human diseases, including cancer [22,23,24,25]. Copper-containing anticancer drugs have been developed that show astonishing activity in the fight against this disease [26,27,28,29], and it can be expected that copper-containing regimens will pass clinical trials in the drug developmental process for new cancer treatments [30,31]. Given the importance of ferroptosis in cancer, the role of ferroptosis and ferroptosis-related genes as a prognostic factor for the survival of patients has been investigated and established for several cancer types, e.g., melanoma, glioma, hepatocellular carcinoma, breast cancer, and others [32,33,34,35].

Cuproptosis is coming up as a new metal ion-dependent cell death mechanism, and copper ionophores are attracting great interest in cancer therapy. Copper-based nanomaterials in bladder cancer have shown improved efficacy to immunotherapy. Cuproptosis-related genes are also showing predictive capacity for patients in various cancers and may help in patient sensitivity to chemotherapy. Although cuproptosis has been investigated recently, less is known about its prognostic role for cancer patient survival [36,37]. Multi-“omics” studies have shown the potential importance of certain genes that mediate cuproptosis as determinants of patient outcomes, and further studies focusing on copper-related gene signatures will offer substantial potential for advancing our understanding of cancer biology.

In the present investigation, we systematically investigated 124 cuproptosis-related genes in more than 7489 tumor biopsies of different tumor types of the Cancer Genome Atlas (TDGA) regarding their mRNA expression and statistical relationship to the survival times of patients by using Kaplan–Meier statistics. Those genes with significant relationships to the survival times of patients were subjected to Ingenuity Pathway Analysis (IPA) to detect their interactions among each other in signaling networks.

## 2. Materials and Methods

### 2.1. Compilation of Cuproptosis-Associated Genes

We screened the PubMed Literature database with the keywords “gene” and “cuproptosis”. Papers containing compilations of cuproptosis-associated genes were used to compile our own list of genes. Cuproptosis-related genes have been identified from the literature and served as the basis for our investigations. These genes are experimentally validated to contribute to the cuproptosis mode of programed cell death [38,39,40,41,42,43,44,45,46,47]. A list containing 124 cuproptosis-related genes is provided in Supplementary Table S1.

The expression of these genes in 7489 tumor biopsies was reported by the Cancer Genome Atlas (TCGA) [38]. The Cancer Genome Atlas (TCGA) is a large genomic program initiated by the National Cancer Institute and the National Human Genome Research Program (Bethesda, MD, USA) to characterize the molecular architecture of a large number of different cancer types by analyzing thousands of tumor samples with different genomic technologies (www.cancer.gov/ccg/research/genome-sequencing/tcga) (accessed on 1 October 2024). Among gene copy number analysis, methylation, and mutational status of DNA, the RNA expression is analyzed. Microarray hybridization and high-throughput sequencing are used for characterizing DNA and RNA. Based on transcriptomic profiling, which comprises all expressed genes of the human genome, subgroups of interest can be formed, e.g., genes driving programmed cell death [48], genes determining anticancer drug response [49], genes related to tumor immunology [50], etc. Previous investigations on cell death focused on apoptosis [51], autophagy [52], and ferroptosis [53]. Therefore, we focused on cuproptosis as novel mode of programmed cell death and selected genes known from the literature as determinants of cuproptosis (see above). The genes selected from the literature were used to run the TCGA-based KM plotter (see below).

### 2.2. Kaplan–Meier Survival Statistics

Based on the TCGA database, the transcriptome-wide expression profiles of cancer samples can be correlated with clinical parameters of the corresponding patients, e.g., their survival times. The connection of molecular and clinical parameters in a common database offers a unique opportunity for advanced biostatistical exploration. In this context, a Kaplan–Meier (KM) plotter platform has been generated that allows the analysis of mRNA expression of single or multiple genes from large datasets. This method allows the identification of novel prognostic markers because of huge datasets in a unified database [54,55]. The analysis of survival times of patients represents a long-lasting and classical method in clinical oncology for many decades. The coupling of Kaplan–Meier-based survival analyses with transcriptomic data facilitates the discovery of novel biomarkers to better understand the determinants of tumor diseases and to further improve cancer treatments by the development of novel target-directed drugs. In the present investigation, we used the KM plotter algorithm (https://kmplot.com/analysis/) (accessed on 1 October 2024) as previously published [56,57]. To avoid type I errors of multiple comparisons, we used false discovery rate corrections [58] with a cut-off of 5%. The database of the KM plotter consists of 7489 biopsies from 20 different tumor types from TCGA.

### 2.3. Ingenuity Pathway Analysis

Ingenuity Pathway Analysis (IPA) (Ingenuity Systems, Qiagen, Redwood City, CA, USA; version: fall release 2023) is a bioinformatic tool to analyze and interpret complex biological data in the context of molecular pathways and cellular networks. We utilized IPA to identify cellular functions, canonical pathways, and individual interaction networks of those cuproptosis-related genes that significantly correlated with any of the 20 tumor types analyzed by Kaplan–Meier statistics (see Section 2.2). Thereby, we constructed interaction networks for cuproptosis based on the survival times of cancer patients.

## 3. Results

**Figure 1:**
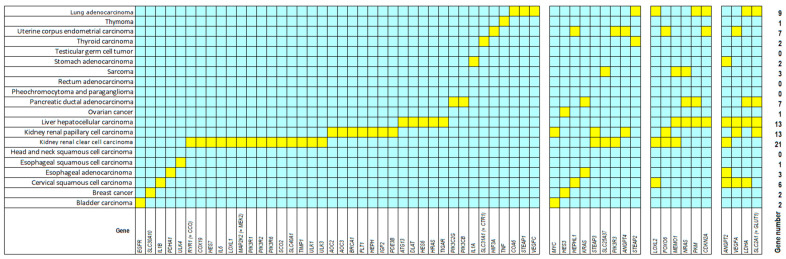
Color-coded plot of Kaplan–Meier statistics of 59 cuproptosis-related genes for 20 tumor types. The yellow color indicates significant correlations between high mRNA expression in tumors and worse overall patient survival (*p* < 0.05; FDR ≤ 5%). The blue boxes show non-significant relationships.

As a starting point, we assembled 124 genes reported in the literature to be associated with the copper-related mode of programed cell death, termed cuproptosis. The mRNA expression of these genes from 7489 biopsies belonging to 20 tumor types have been subjected to Kaplan–Meier statistics using the KM plotter algorithm. A total of 63 out of 124 genes significantly correlated with the outcome of the disease, i.e., high expression of these genes in the tumors was associated with shorter overall survival of patients at a significance level of *p* < 0.05 and a false discovery rate (FDR) of ≤5%. The results are shown in Supplementary Table S1 and illustrated in the plot depicted in Figure 1. Here, the yellow boxes indicate the significant correlations (*p* = 0.05; FDR ≤ 5%) between gene expression and survival times in the different tumor types, while blue boxes show the non-significant relationships. The genes on the left side of Figure 1 show significant associations to each one tumor type (*n* = 40), the genes in the middle to two, three, or four tumor entities (*n* = 9, *n* = 6, and *n* = 4), respectively, and the genes at the right side to four cancer types (*n* = 4).

**Figure 2:**
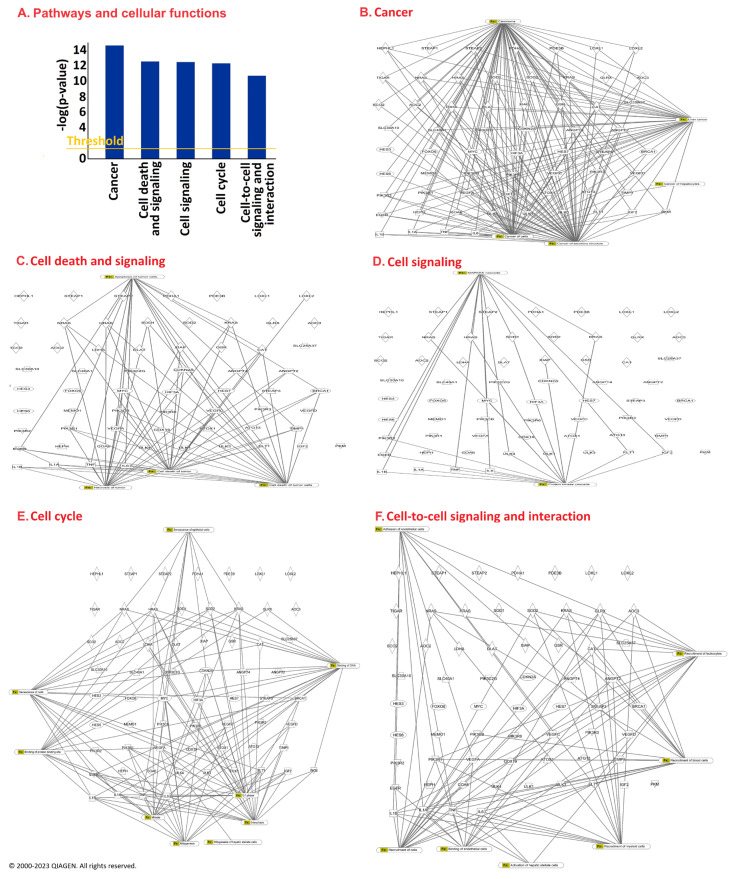
Ingenuity Pathway Analysis of cuproptosis-associated genes that significantly correlated with overall survival of cancer patients. The canonical pathway analysis in the top left panel shows the top five pathways identified. The corresponding networks of these canonical pathways are shown in the other panels of this figure.

As all these gene expressions correlated with the survival times of at least one tumor type, we applied Ingenuity Pathway Analysis (IPA) software (version: fall release 2023 to construct interaction networks with the purpose of obtaining comprising signaling pathways for the cuproptotic mode of cell death. The top five pathways are shown in Figure 2. They included cancer, cell death and cell signaling, cell signaling, cell cycle, and cell-to-cell signal and interaction. Importantly, these pathways are linked to the progression and prognosis of cancer patients. Cancer cells progress through the cell cycle in an uncontrolled manner, implying that a malfunctioning cell cycle contributes to cellular malignancy. This process is connected to defects in signaling pathways that contribute to the homeostasis in normal cells but favor abnormal cell growth, invasion, and metastasis in tumor cells. It was interesting to observe that the genes we identified by IPA as prognostic relevant belonged to pathways that were not only involved in cellular functions crucial for cancer biology in general (i.e., cancer, cell signaling, cell signaling and interaction) but also in the fate of cancer cells upon treatment (i.e., cell death and survival, cell cycle) (Figure 2A). The individual networks for how these genes were predicted to interact with each other are shown in Figure 2B–F.

Counting the number of genes correlating with various tumor types revealed that the expression of 39 out of 63 genes correlated with survival times of patients with only four tumor types, i.e., renal clear cell carcinoma (KIRC), renal papillary cell carcinoma (KIRP), hepatocellular carcinoma (LIHC), and lung adenocarcinoma (LUAD). These genes and their functions are compiled in Table 1. The number of correlating genes was lower in all other tumor types investigated.

**Figure 3:**
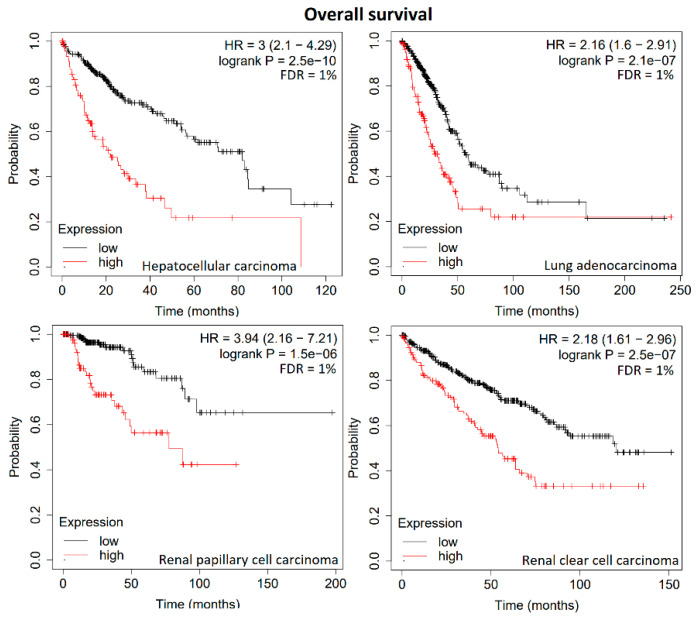
Kaplan–Meier statistics of overall survival times of patients with hepatocellular carcinoma, lung adenocarcinoma, renal papillary cell carcinoma, or renal clear cell carcinoma. The mean mRNA expression of a 13-gene signature has been subjected to hepatocellular carcinoma, of a 9-gene signature to lung adenocarcinoma, of a 13-gene signature to renal papillary, and of a 21-gene signature to renal clear cell carcinoma.

Therefore, we used the mean expression levels of these groups of genes to investigate the prognostic value for these four tumor types, i.e., we calculated the mean mRNA expression of 21 genes in renal clear cell carcinoma, each of the 13 genes in papillary cell carcinoma and hepatocellular carcinoma, and 9 genes in lung adenocarcinoma. As expected, the Kaplan–Meier survival curves were statistically significant, with very low *p*-values ranging from 1.5 × 10^−6^ to 2.5 × 10^−10^ and FDR values of 1% (Figure 3). This indicates that these group of genes represent gene signatures with high prognostic value for patients suffering from these four tumor types.

To investigate not only overall survival times, we also studied the prognostic value of these gene signatures for refractory-free survival times, but the set criteria (*p* < 0.05; FDR ≤ 5%) were not reached for any of the four tumor entities.

**Figure 4:**
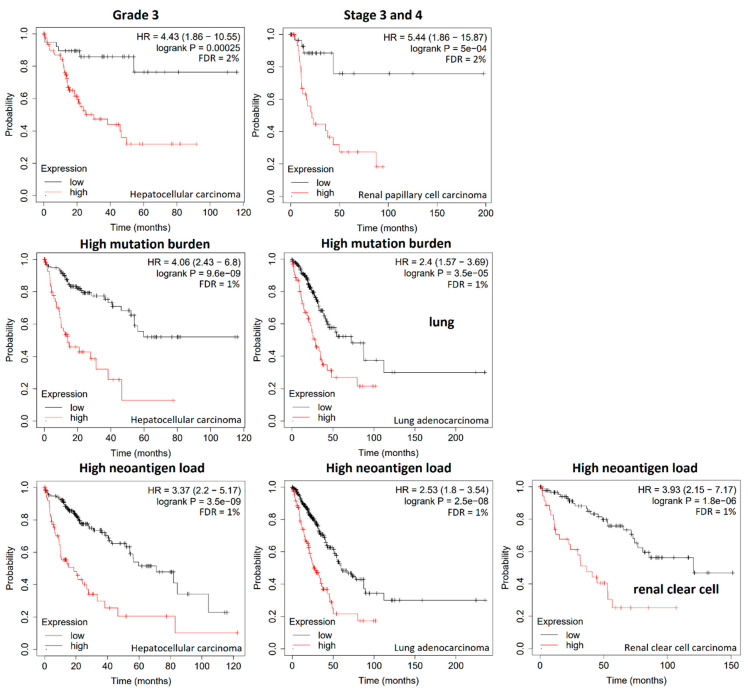
Kaplan–Meier statistics of overall survival times of patients with clinical parameters (grade 3, stages 3 and 4) and molecular parameters of high mutation burden and high neoantigen load. Survival curves are shown for hepatocellular carcinoma, renal papillary cell carcinoma, lung adenocarcinoma, and renal clear cell carcinoma. The mean mRNA expressions of the respective gene signature were subjected for survival time analyses.

As the survival curves in Figure 3 result from all tumors of the respective tumor types, we were interested to see the relevance of these gene signatures in subgroups, i.e., tumors of grade 3 or in stage 3 and 4 as well as tumors with high mutation burden or high neoantigen load because these parameters also influence the treatment outcome. The results of significant correlations in these clinical and molecular subgroups are shown in Figure 4. The expression of the 13-gene signature was significantly correlated with the survival times of patients with hepatocellular carcinoma grade 3 (Figure 4, top panel, left), while the gene signatures of the other three tumor types did not yield significant results regarding tumor grading. The 21-gene signature of renal clear cell carcinoma correlated with survival of stage 3 and four tumor types (Figure 4, top panel, right). Significant correlations were not found to other tumor types in stages 3 and 4. Furthermore, we investigated the relationships of gene expression and high mutation burden. The respective gene signatures significantly correlated with survival times of hepatocellular carcinoma and lung adenocarcinoma with high mutational burden (Figure 4, middle panel) but not of renal clear cell carcinoma or renal papillary cell carcinoma. Finally, the neoantigen load of tumors was subjected to Kaplan–Meier analyses. High neoantigen load of hepatocellular carcinoma, lung adenocarcinoma, and renal clear cell carcinoma patients correlated with the corresponding gene signatures (Figure 4, bottom panel). A significant relationship to renal papillary cell carcinoma was not found.

## 4. Discussion

Cuproptosis represents a novel mode of cell death involving copper [18,20]. This transition metal plays a crucial role in normal physiology under healthy conditions but also in the pathophysiology of many diseases [21,22,24]. Since cuproptosis is also a relevant mechanism of non-apoptotic cell death in cancer [25], we focused on the prognostic relevance of cuproptosis in cancer using the TCGA dataset. We found that at least some cuproptosis-relevant genes exert prognostic significance in all 20 different tumor types analyzed. Interestingly, however, the expression of cuproptosis-related genes was clustered in four tumor types (hepatocellular carcinoma, renal clear cell carcinoma, papillary renal cell carcinoma, and lung adenocarcinoma), indicating that cuproptosis may play an important role in these tumor types. Our results highlight (1) a 13-gene signature and its correlation with survival times for hepatocellular carcinoma grade 3 patients; (2) another 21-gene signature and its correlation to the survival times of stage 3 and 4 renal clear cell carcinomas; and (3) the gene signatures correlating with high mutational burden and neoantigen load of lung adenocarcinoma, hepatocellular carcinoma, and renal clear cell carcinoma patients, respectively. Indeed, the relevance of copper has been previously documented in these tumor types [59,60,61], and our study gives more insight into this importance.

The quest for prognostic markers indicating survival probabilities has been at the center of interest in clinical oncology since the early days. While clinicopathological parameters (such as tumor size, lymph node and distant metastasis, and tumor differentiation) represent classical categories in this context, this armory has been supplemented during the past three decades by molecular markers and recently by transcriptome-wide RNA-sequencing [62,63,64]. The latter one especially allows one to simultaneously identify not only single but multiple prognostic factors in a comprising manner in a short time. In the present investigation, we took advantage of the TCGA, which assembled one of the largest data collections in the history of clinical oncology. The systematic evaluation of cuproptosis-related genes performed by us unraveled clusters (or call them signatures) of multiple genes whose expression was significantly correlated with the survival times of patients. We identified gene signatures consisting of nine to twenty-one genes in four tumor types. Other authors also reported gene signatures with varying numbers of genes with prognostic relevance for renal clear cell carcinoma [38,65,66], hepatocellular carcinoma [51,52,53], and lung adeno carcinoma [39,67,68,69,70,71]. Gene signatures have not been reported for papillary cell carcinoma yet. Interestingly, these gene signatures did not only predict survival probabilities but also detected subtypes of tumors that otherwise appeared homogenous upon histopathological examination. The further formation of new subtypes of cancers makes the prediction of patients’ survival more precise. These signatures maybe all used to develop novel clinical routine diagnostics to predict the survival chances of individual patients in the future. Hence, our approach may further contribute to set up new tools for the individualization and improved precision of cancer medicine.

As shown here, these gene expression signatures can not only be combined with classical prognostic parameters (such as tumor stage and grade) but also with new ones (such as gene mutation rates and neoantigen load) to further enhance prognostic power. The mutation rate is a parameter for genetic instability and tumor progression, and thereby provides the possibility to apply novel targeted drugs addressing mutated driver genes in carcinogenesis. The neoantigen load may provide information on the utility of immunotherapies specifically attacking newly appearing tumor markers that are absent in the corresponding normal tissues the tumors are derived from [72].

The identification of novel prognostic factors and signatures as new diagnostic tools in clinical oncology are only one side of the coin. Another question relates to their function and interaction with each other. Since the description of network pharmacology as a new concept in pharmacology by Shao Li and Andrew L. Hopkins [73,74,75,76], it has become increasingly clearer that complex networks of interacting proteins drive tumor progression and determine treatment outcome. Therefore, studying the complex networks represents another important task that we addressed in the present investigation.

The interaction networks constructed based on prognostically relevant genes give interesting insights into the mechanisms that drive tumor progression and, hence, influence the survival of patients. The functions of proteins encoded by the identified genes could be basically assigned to the following six main mechanisms:

- Cell growth and gene expression (*CDKN2A, FOXO6, HES6, HES7, IGF2, LOXL1, MEMO1, TIMP1*);
- Oncogenes and tumor suppressors (*BRCA1, HRAS, NRAS*);
- Signal transduction (*MAP2K2, PIK3R1, PIK3R2, PIK3R3, PIK3R6, ULK1, ULK6*);
- Angiogenesis (*ANGPT4, FLT1, PDE3B, VEGFC*);
- Metabolism (*AOC2, AOC3, COA6, DLAT, PKM, SCO2, TIGAR*);
- Transporters and channels (*HEPH, RYR1, SCL2A1, SLC40A1, STEAP2*).

These mechanisms represent basic features of cancer cells. The deregulation of oncogenes and tumor suppressor genes as well as cancer-specific metabolic changes and signal transduction are tightly connected with altered signal transduction and transporter and ion channel functions, ultimately leading to cancer cell growth and tumor neoangiogenesis. The interweaving of these mechanisms may provide a possible explanation for the prognostic role of these genes regarding patients’ survival. We have visualized these interactions by using the IPA tool. Pathway analyses are highly valuable for the elucidation of proteomic and transcriptomic data to generate hypotheses for the complex interaction networks of proteins and genes in diseased cells and tissues. We used this technique as an approach to generate interaction maps of genes with prognostic significance. Such interaction maps emphasize the fact that the orchestration of multiple rather than single genes determines tumor progression and, finally, the fate of patients.

As can be expected, not all these cuproptosis-related genes contribute to the survival chances of cancer patients. Therefore, we performed Kaplan–Meier analyses and found that several genes significantly correlated with each of the 20 tumor types included in the investigation. Then, we focused on the four tumor types with the highest number of correlating genes. It is a well-known approach in the literature to focus not only on single genes but on entire groups of genes, so-called signatures. The idea behind this approach is that gene signatures may predict the survival chances with higher precision than single genes. Gene signatures with prognostic value for the survival of patients have been described for various cancer types, including the most common cancer types, such as breast cancer, lung cancer, prostate cancer, etc. [77,78,79,80,81]. Here, we identified gene signatures for four tumor entities, i.e., renal papillary cell carcinoma, renal clear cell carcinoma, hepatocellular carcinoma, and lung adenocarcinoma, with a specific focus on one mode of cell death, cuproptosis, and combined this point of view with other prognostic factors such as stage and grade, as well as molecular markers such as mutation rate and neoantigen load.

The task for the future would be to compare the diverse genes signatures reported In the literature and to delineate consensus gene signatures that can be developed for diagnostic test systems to predict the survival probabilities of cancer patients.

## 5. Conclusions

Kaplan–Meier statistics in 7489 biopsies from 20 different tumor types revealed that the high mRNA expression of 63/124 cuproptosis-associated genes significantly correlated with shorter survival times of cancer patients. The accumulation of significantly correlating cuproptosis-related genes in renal clear cell carcinoma, renal papillary carcinoma, hepatocellular carcinoma, and lung adenocarcinoma indicated that this mode of cell death may play an important role in these four tumor types. The relevance of these genes as prognostic markers in clinical routine diagnostics needs to be further explored in the future by validation of identified gene signatures in clinical settings or comparison with other reported gene signatures.

## Figures and Tables

**Table 1:** Prognostic significance of mRNA expression of selected genes for overall survival of patients with hepatocellular carcinoma, lung adenocarcinoma, renal clear cell carcinoma, or renal papillary carcinoma.

Symbol	Gene Name	Function	Tumor Type	Sample No.	*p*-Value	FDR
*RYR1 (=CCO)*	Ryanodine receptor 1 (skeletal)	Calcium release channel	KIRC	530	2.1 × 10^−4^	5%
*HES7*	Hairy and enhancer of split (*Drosophila*) family BHLH transcription factor 7	Transcriptional repressor	KIRC	530	6.7 × 10^−7^	1%
*IL6*	Interleukin 6	Proinflammatory cytokine	KIRC	530	5.3 × 10^−8^	1%
*LOXL1*	Lysine oxidase-like	Biogenesis of connective tissue	KIRC	530	3.0 × 10^−7^	1%
*MAP2K2* *(=MEK2)*	Mitogen-activated protein kinase kinase 2	Mitogenic growth factor, signal transduction	KIRC	530	3.7 × 10^−4^	1%
*PIK3R1*	Phosphoinositide-3-kinase regulatory subunit 1	Role in the metabolic insulin action	KIRC	530	4.9 × 10^−5^	1%
*PIK3R2*	Phosphoinositide-3-kinase regulatory subunit 2	Regulatory component of PI3K, growth signaling pathways	KIRC	530	1.0 × 10^−4^	3%
*PIK3R6*	Phosphoinositide-3-kinase regulatory subunit 6	Regulatory component of PI3K, growth signaling pathways	KIRC	530	5.3 × 10^−5^	1%
*SCO2*	Synthesis of cytochrome C oxidase 1	Role in aerobic ATP production	KIRC	530	1.6 × 10^−4^	1%
*SLC40A1*	Solute carrier family 40 member 1	Iron export	KIRC	530	8.5 × 10^−13^	1%
*TIMP1*	Tissue inhibitor of metalloproteinases 1	Degradation of extracellular matrix, cell proliferation	KIRC	530	2.1 × 10^−4^	1%
*ULK1*	Unc-51-like autophagy- activating kinase 1	Serine/threonine kinase, autophagosome assembly	KIRC	530	5.6 × 10^−6^	1%
*ULK3*	Unc-51-like autophagy- activating kinase 3	Serine/threonine kinase, fibroblast activation	KIRC	530	1.0 × 10^−4^	3%
*AOC2*	Amine oxidase copper- containing 2	Oxidative conversion of amines to aldehydes and ammonia	KIRP	287	3.0 × 10^−4^	5%
*AOC3*	Amine oxidase copper- containing 3	Adhesive properties, leukocyte trafficking	KIRP	287	1.6 × 10^−4^	2%
*BRCA1*	Breast and ovarian cancer susceptibility protein 1	Tumor suppressor	KIRP	287	4.1 × 10^−5^	1%
*FLT1*	Fms-related receptor tyrosine kinase 1	Role in angiogenesis	KIRP	287	4.1 × 10^−6^	1%
*HEPH*	Hephaestin	Copper and iron transport and homeostasis	KIRP	287	5.0 × 10^−4^	5%
*IGF2*	Insulin-like growth factor 2	Cell development and growth	KIRP	287	7.2 × 10^−5^	1%
*PDE3B*	Phosphodiesterase 3B	Negative regulation of angiogenesis and cell adhesion	KIRP	287	2.4 × 10^−4^	3%
*ATG13*	Autophagy-related 13	Autophagosome formation and mitophagy	LIHC	370	6.0 × 10^−5^	1%
*DLAT*	Dihydrolipoamide S-acetyltransferase	Component of the pyruvate dehydrogenase complex	LIHC	370	5.0 × 10^−5^	1%
*HES6*	Hairy and enhancer of split (*Drosophila*) family BHLH transcription factor 6	Regulation of cell differentiation	LIHC	370	2.1 × 10^−4^	3%
*HRAS*	Harvey rat sarcoma viral oncogene homolog	Oncogenic GTPase	LIHC	370	2.3 × 10^−4^	5%
*TIGAR*	TP53-induced glycolysis regulatory phosphatase	Blockage of glycolysis	LIHC	370	3.2 × 10^−4^	5%
*COA6*	Cytochrome c oxidase assembly factor 6	Mitochondrial respiration	LUAD	504	1.1 × 10^−4^	3%
*STEAP1*	Six-transmembrane epithelial antigen of prostate metalloreductase 1	Cell surface antigen at cell–cell junctions	LUAD	504	1.4 × 10^−6^	1%
*VEGFC*	Vascular endothelial growth	Angiogenesis and endothelial	LUAD	504	3.0 × 10^−4^	1%
	factor C	cell growth	KIRP	287	4.1 × 10^−4^	5%
*STEAP3*	STEAP3 metalloreductase	Iron and copper transporter	KIRC	530	3.7 × 10^−2^	1%
		in p53-mediated apoptosis	KIRP	287	1.3 × 10^−5^	1%
*SLC25A37*	Solute carrier family 25 member 37	Imports iron for the synthesis of mitochondrial heme	KIRP	530	3.3 × 10^−7^	1%
*PIK3R3*	Phosphoinositide-3-kinase regulatory subunit 3	Second messenger in growth signaling pathways	KIRC	530	4.6 × 10^−8^	1%
*ANGPT4*	Angiopoietin 4	Involved in angiogenesis	KIRP	287	3.1 × 10^−4^	5%
*STEAP2*	STEAP2 metalloreductase	Iron and copper uptake	LUAD	504	1.2 × 10^−4^	3%
			KIRC	530	3.5 × 10^−5^	1%
			LUAD	504	3.7 × 10^−5^	1%
*FOXO6*	Forkhead box O6	Regulation of transcription	KIRC	530	5.4 × 10^−5^	1%
		by RNA polymerase II	KIRP	287	3.6 × 10^−4^	5%
*MEMO1*	Mediator of cell motility 1	Microtubule-based processes	KIRC	530	3.3 × 10^−7^	1%
			LIHC	370	1.7 × 10^−4^	3%
*NRAS*	Neuroblastoma RAS viral oncogene homolog	Oncogenic GTPase	LIHC	370	3.4 × 10^−5^	1%
*PKM*	Pyruvate kinase M	Glycolysis	LIHC	370	2.7 × 10^−6^	1%
			LUAD	504	1.5 × 10^−4^	3%
*CDKN2A*	Cyclin-dependent kinase	Regulation of the G1 phase of	LIHC	370	2.2 × 10^−4^	5%
	inhibitor 2A	the cell cycle	LUAD	504	2.4 × 10^−4^	5%
			KIRP	287	7.1 × 10^−8^	1%
			LIHC	370	6.3 × 10^−5^	1%
			KIRP	287	1.6 × 10^−7^	1%
			LIHC	370	2.3 × 10^−5^	1%
			LIHC	370	3.9 × 10^−7^	1%
			LUAD	504	1.8 × 10^−6^	1%
*SLC2A1*	Glucose transporter type 1	Glucose transport in the blood	KIRP	287	4.5 × 10^−4^	5%
*(=GLUT1)*		brain barrier	LIHC	370	2.7 × 10^−8^	1%
			LUAD	504	3.6 × 10^−7^	1%

## Data Availability

The data are available upon reasonable request.

## Supplementary Materials

The following supporting information can be downloaded at: https://www.mdpi.com/article/10.3390/biology13100793/s1. Table S1: Kaplan–Meier overall survival analysis of 124 genes associated with the cuproptosis type of programed cell death in 7489 biopsies of The Cancer Genome Atlas (TCGA) as recently compiled [48,52,66,67,68,69,70,71,72,73,74].
