# Supplementary material for: Identification of Cuproptosis-Associated Prognostic Gene Expression Signatures from 20 Tumor Types
Source: Biology (Basel). 2024 Oct 3;13(10):793. doi: 10.3390/biology13100793 (PMC11505359; doi:10.3390/biology13100793)
Supplement: Supplementary file 1 [file biology-13-00793-s001.zip › biology-3115300-supplementary.pdf]

Supplementary Table S1: Kaplan-Meier overall survival analysis of 124 genes associated with the cuproptosis type of programed cell death in 7489 biopsies of The Cancer Genome Atlas (TCGA) as recently compiled [48,52,66-74].

No.	Symbol	Gene Name	Tumor type	Sample number	P-Value	FDR
1	AKT	V-Akt murine thymoma viral oncogene homolog 1	none			
2	ANGPT	Angiopoietin	none			
3	ANGPT1	Angiopoietin 1	none			
4	ANGPT2	Angiopoietin 2	Cervical squamous cell carcinoma	304	6.2×10^{-5}	1%
			Esophageal Adenocarcinoma	80	5.3×10^{-4}	2%
			Kidney renal papillary cell carcinoma	287	7.1×10^{-8}	1%
			Liver hepatocellular carcinoma	370	6.3×10^{-5}	1%
			Stomach adenocarcinoma	371	2.9×10^{-4}	5%
5	ANGPT4	Angiopoietin 4	Kidney renal papillary cell carcinoma	287	3.1×10^{-4}	5%
			Uterine corpus endometrial carcinoma	542	7.7×10^{-5}	2%
6	AOC1	Amine oxidase copper-containing 1	none			
7	AOC2	Amine oxidase copper-containing 2	Kidney renal papillary cell carcinoma	287	3.0×10^{-4}	5%
8	AOC3	Amine oxidase copper-containing 3	Kidney renal papillary cell carcinoma	287	1.6×10^{-4}	2%
9	ATG13	Autophagy-related 13	Liver hepatocellular carcinoma	370	6.0×10^{-5}	1%
10	ATP7A	ATPase copper-transporting α	none			
11	ATP7B	TablATPase copper-transporting β	none			
13	BRCA1	Breast and ovarian cancer susceptibility protein 1	Kidney renal papillary cell carcinoma	287	4.1×10^{-5}	1%
15	CCS	Cu chaperone for superoxide dismutase	none			
16	CDKN2A	Cyclin-dependent kinase inhibitor 2A	Liver hepatocellular carcinoma	370	2.2×10^{-4}	5%
			Lung adenocarcinoma	504	2.4×10^{-4}	5%
			Uterine corpus endometrial carcinoma	542	1.8×10^{-7}	1%
17	COA6	Cytochrome c oxidase assembly factor 6	Lung adenocarcinoma	504	1.1×10^{-4}	3%
18	COMMD1	Copper metabolism domain-containing 1	none			
19	COX11	Cytochrome C oxidase copper caperone 11	none			
20	COX17	Cytochrome c oxidase copper chaperone 17	none			
21	COX19	Cytochrome C oxidase copper chaperone 19	Kidney renal clear cell carcinoma	530	3.6×10^{-8}	1%

22	CP	Ceruloplasmin	none			
23	CUTC	CutC copper transporter	none			
24	DLAT	Dihydrolipoamide S-acetyltransferase	Liver hepatocellular carcinoma	370	5.0×10^{-5}	1%
25	DLD	Dihydrolipoamide dehydrogenase	none			
26	EGFR	Epidermal growth factor receptor	Bladder carcinoma	404	3.8×10^{-5}	1%
27	FDX1	Ferredoxin 1	none			
28	FDXR	Ferredoxin reductase	none			
29	FGF2	Basic fibroblast growth factor	none			
30	FLT1	Fms-related receptor tyrosine kinase 1	Kidney renal papillary cell carcinoma	287	4.1×10^{-6}	1%
31	FOXO1	Forkhead box O1	none			
12	FOXO3	Forkhead box O3	none			
32	FOXO4	Forkhead box O4	none			
33	FOXO6	Forkhead box O6	Kidney renal clear cell carcinoma	530	5.4×10^{-5}	1%
			Kidney renal papillary cell carcinoma	287	3.6×10^{-4}	5%
			Uterine corpus endometrial carcinoma	542	8.2×10^{-6}	1%
34	FXN	Frataxin	none			
35	GLS	Glutaminase	none			
36	GLS2	Glutaminase 2	none			
37	HEPH	Hephaestin	Kidney renal papillary cell carcinoma	287	5.0×10^{-4}	5%
38	HEPHL1	Hephaestin-like 1	Cervical squamous cell carcinoma	304	4.6×10^{-4}	5%
			Uterine corpus endometrial carcinoma	542	4.2×10^{-6}	1%
39	HES1	Hairy and enhancer of split (*Drosophila*) family BHLH transcription factor 1	none			
41	HES2	Hairy and enhancer of split (*Drosophila*) family BHLH transcription factor 2	none			
42	HES3	Hairy and enhancer of split (*Drosophila*) family BHLH transcription factor 3	Breast cancer	1089	4.4×10^{-7}	1%
			Ovarian cancer	373	2.9×10^{-6}	1%
43	HES4	Hairy and enhancer of split (*Drosophila*) family BHLH transcription factor 4	none			
44	HES5	Hairy and enhancer of split (*Drosophila*) family BHLH transcription factor 5	none			
45	HES6	Hairy and enhancer of split (*Drosophila*) family BHLH transcription factor 6	Liver hepatocellular carcinoma	370	2.1×10^{-4}	3%

	HES7	Hairy and enhancer of split (*Drosophila*) family BHLH transcription factor 7	Kidney renal clear cell carcinoma	530	6.7×10^{-7}	1%
46	HEY1	Hairy ears, Y-linked 1	none			
47	HEY2	Hairy ears, Y-linked 2	none			
48	HIF1A	Nuclear hypoxia inducible factor-1 α	none			
49	HIF3A	Nuclear hypoxia inducible factor-1 α	Uterine corpus endometrial carcinoma	542	2.0×10^{-7}	1%
50	HRAS	Harvey rat sarcoma viral oncogene homolog	Liver hepatocellular carcinoma	370	2.3×10^{-4}	5%
51	IGF1	Insulin-like growth factor 1	none			
52	IGF2	Insulin-like growth factor 2	Kidney renal papillary cell carcinoma	287	7.2×10^{-5}	1%
53	IL1A	Interleukin 1α	Stomach adenocarcinoma	371	3.8×10^{-4}	5%
54	IL1B	Interleukin 1β	Cervical squamous cell carcinoma	304	1.4×10^{-4}	2%
55	IL6	Interleukin 6	Kidney renal clear cell carcinoma	530	5.3×10^{-8}	1%
56	ISCU	Iron-sulfur cluster assembly enzyme	none			
57	MAPK8 (= JNK)	C-Jun N-terminal kinase 1	none			
58	KRAS	Kirsten rat sarcoma viral oncogene homolog	Esophageal Adenocarcinoma	80	9.0×10^{-4}	3%
			Pancreatic ductal adenocarcinoma	177	3.6×10^{-4}	3%
59	LDHA	Lactate dehydrogenase A	Cervical squamous cell carcinoma	304	7.8×10^{-5}	1%
			Liver hepatocellular carcinoma	370	3.9×10^{-7}	1%
			Lung adenocarcinoma	504	1.8×10^{-6}	1%
			Pancreatic ductal adenocarcinoma	177	8.0×10^{-6}	1%
60	LIAS	Lipoic acid synthetase	none			
61	LIPT1	Lipoyltransferase 1	none			
62	LOX	Lysine oxidase	none			
63	LOXL1	Lysine oxidase-like	Kidney renal clear cell carcinoma	530	3.0×10^{-7}	1%
64	LOXL2	Lysine oxidase-like 2	Cervical squamous cell carcinoma	304	9.9×10^{-6}	1%
			Kidney renal clear cell carcinoma	530	3.5×10^{-5}	1%
			Lung adenocarcinoma	504	3.7×10^{-5}	1%
65	LOXL3	Lysine oxidase-like 3	none			
66	LOXL4	Lysine oxidase-like 4	none			
67	MAP2K1 (= MEK1)	Mitogen-activated protein kinase kinase 1	none			
68	MAP2K2 (= MEK2)	Mitogen-activated protein kinase kinase 2	Kidney renal clear cell carcinoma	530	3.7×10^{-7}	1%
69	MAP2K4 (= MEK4)	Mitogen-activated protein kinase kinase 4	none			
70	MEMO1	Mediator of cell motility 1	Kidney renal clear cell carcinoma	530	3.3×10^{-7}	1%
			Liver hepatocellular carcinoma	370	1.7×10^{-4}	3%

			Sarcoma	259	1.1×10^{-5}	1%
71	MTF1	Metal-regulatory transcription factor 1	none			
72	mTOR	Mammalian mechanistic target of rapamycin kinase	none			
73	MYC	Avian myelocytomatosis viral oncogene homolog	Bladder Carcinoma	404	5.8×10^{-5}	1%
			Kidney renal papillary cell carcinoma	287	4.1×10^{-4}	5%
74	NRAS	Neuroblastoma RAS viral oncogene homolog	Liver hepatocellular carcinoma	370	3.4×10^{-5}	1%
			Pancreatic ductal adenocarcinoma	177	3.3×10^{-4}	3%
			Sarcoma	259	4.8×10^{-4}	5%
75	PDE3B	Phosphodiesterase 3B	Kidney renal papillary cell carcinoma	287	2.4×10^{-4}	3%
76	PDHA1	Pyruvate dehydrogenase E1 subunit α1	Esophageal Adenocarcinoma	80	3.7×10^{-4}	2%
77	PDHB	Pyruvate dehydrogenase E1 subunit β	none			
78	PDK1	Pyruvate Dehydrogenase Kinase 1	none			
79	PIK3C2A	Phosphatidylinositol 3-kinase catalytic subunit type 2A	none			
80	PIK3C2B	Phosphatidylinositol 3-kinase catalytic subunit type 2B	none			
81	PIK3C2G	Phosphatidylinositol 3-kinase catalytic subunit type 2G	Pancreatic ductal adenocarcinoma	177	3.5×10^{-4}	3%
82	PIK3C3	Phosphatidylinositol 3-kinase catalytic subunit type 3	none			
83	PIK3CA	Phosphatidylinositol 3-kinase catalytic subunit type A	none			
84	PIK3CB	Phosphatidylinositol 3-kinase catalytic subunit type B	Pancreatic ductal adenocarcinoma	177	4.5×10^{-4}	5%
85	PIK3CD	Phosphatidylinositol 3-kinase catalytic subunit type D	none			
86	PIK3R1	Phosphoinositide-3-kinase regulatory subunit 1	Kidney renal clear cell carcinoma	530	4.9×10^{-5}	1%
87	PIK3R2	Phosphoinositide-3-kinase regulatory subunit 2	Kidney renal clear cell carcinoma	530	1.0×10^{-4}	3%
88	PIK3R3	Phosphoinositide-3-kinase regulatory subunit 3	Kidney renal clear cell carcinoma	530	4.6×10^{-8}	1%
			Uterine corpus endometrial carcinoma	542	1.9×10^{-4}	5%
89	PIK3R4	Phosphoinositide-3-kinase regulatory subunit 4	none			

90	PIK3R5	Phosphoinositide-3-kinase regulatory subunit 5	none			
91	PIK3R6	Phosphoinositide-3-kinase regulatory subunit 6	Kidney renal clear cell carcinoma	530	5.3×10^{-5}	1%
92	PKM	Pyruvate kinase M	Liver hepatocellular carcinoma	370	2.7×10^{-6}	1%
			Lung adenocarcinoma	504	1.5×10^{-4}	3%
			Pancreatic ductal adenocarcinoma	177	1.0×10^{-5}	1%
93	RPS6KB1 (= S6K1)	Ribosomal protein S6 kinase B1	none			
94	RYR1 (= CCO)	Ryanodine receptor 1 (skeletal)	Kidney renal clear cell carcinoma	530	2.1×10^{-4}	5%
95	SCO1	Synthesis of cytochrome c oxidase1	none			
96	SCO2	Synthesis of cytochrome c oxidase1	Kidney renal clear cell carcinoma	530	1.6×10^{-5}	1%
97	SESN1	Sestrin 1	none			
98	SESN2	Sestrin 2	none			
99	SESN3	Sestrin 3	none			
100	SLC11A2 (= DMT1)	Solute carrier family 11 member 2 (= divalent metal transporter 1)	none			
101	SLC2A1 (= GLUT1)	Glucose transporter type 1	Kidney renal papillary cell carcinoma	287	4.9×10^{-4}	5%
			Liver hepatocellular carcinoma	370	2.7×10^{-8}	1%
			Lung adenocarcinoma	504	3.6×10^{-7}	1%
			Pancreatic ductal adenocarcinoma	177	4.4×10^{-5}	1%
102	SLC25A3	Solute carrier family 25 member 3	none			
103	SLC25A37	Solute carrier family 25 member 37	Kidney renal clear cell carcinoma	530	3.3×10^{-7}	1%
			Sarcoma	259	3.8×10^{-4}	5%
104	SLC30A10	Solute carrier family 30 member 10	Breast cancer	1089	6.1×10^{-5}	3%
105	SLC31A1 (= CTR1)	Solute carrier family 31 member 1 (= copper transporter 1)	Thyroid carcinoma	502	2.3×10^{-4}	5%
106	SLC31A2 (= CTR2)	Solute carrier family 31 member 2 (= copper transporter 2)	none			
107	SLC40A1	Solute carrier family 40 member 1	Kidney renal clear cell carcinoma	530	8.5×10^{-13}	1%
108	STEAP1	Six-transmembrane epithelial antigen of prostate metalloreductase 1	Lung adenocarcinoma	504	1.4×10^{-6}	1%
109	STEAP2	STEAP2 metalloreductase	Lung adenocarcinoma	504	1.2×10^{-4}	3%
			Thyroid carcinoma	502	2.4×10^{-4}	5%
110	STEAP3	STEAP3 metalloreductase	Kidney renal clear cell carcinoma	530	3.7×10^{-12}	1%
			Kidney renal papillary cell carcinoma	287	1.3×10^{-5}	1%

111	STEAP4	STEAP4 metalloreductase	none			
112	TIGAR	TP53-Induced glycolysis regulatory phosphatase	Liver hepatocellular carcinoma	370	3.2×10^{-4}	5%
113	TIMP1	Tissue inhibitor of metalloproteinases 1	Kidney renal clear cell carcinoma	530	2.1×10^{-8}	1%
114	TNF	Tumor necrosis factor	Thymoma	118	7.3×10^{-4}	5%
115	TP53	Tumor suppressor 53	none			
116	ULK1	Unc-51-like autophagy-activating kinase 1	Kidney renal clear cell carcinoma	530	5.6×10^{-6}	1%
117	ULK2	Unc-51-like autophagy-activating kinase 2	none			
118	ULK3	Unc-51-like autophagy-activating kinase 3	Kidney renal clear cell carcinoma	530	1.0×10^{-4}	3%
119	ULK4	Unc-51-like autophagy-activating kinase 4	Esophageal Adenocarcinoma	80	1.4×10^{-4}	5%
120	VEGFA	Vascular endothelial growth factor A	Cervical squamous cell carcinoma	324	4.5×10^{-4}	5%
			Kidney renal papillary cell carcinoma	287	1.6×10^{-7}	1%
			Liver hepatocellular carcinoma	370	2.3×10^{-5}	1%
			Uterine corpus endometrial carcinoma	542	2.1×10^{-4}	5%
121	VEGFB	Vascular endothelial growth factor B	none			
122	VEGFC	Vascular endothelial growth factor C	Lung adenocarcinoma	504	3.0×10^{-5}	1%
123	VEGFD	Vascular endothelial growth factor D	none			
124	XIAP	X-linked inhibitor of apoptosis	none			
